# The KIDSCREEN-27 scale: translation and validation study of the Slovenian version

**DOI:** 10.1186/s12955-022-01973-3

**Published:** 2022-04-21

**Authors:** Leona Cilar Budler, Majda Pajnkihar, Ulrike Ravens-Sieberer, Owen Barr, Gregor Stiglic

**Affiliations:** 1 Faculty of Health Sciences, University of Maribor, Zitna ulica 15, 2000 Maribor, Slovenia; 2 Center for Psychosocial Medicine, University Medical Center Hamburg – Eppendorf, Martinistrasse 52, 20246 Hamburg, Germany; 3 School of Nursing, Ulster University, Northland Road, Londonderry, BT48 7JL UK

**Keywords:** Social support, Adolescent, Psychometrics, Factor analysis, Quality of life

## Abstract

**Background:**

There are many methods available for measuring social support and quality of life (QoL) of adolescents, of these, the KIDSCREEN tools are most widely used. Thus, we aimed to translate and validate the KIDSCREEN-27 scale for the usage among adolescents aged between 10 and 19 years old in Slovenia.

**Methods:**

A cross-sectional study was conducted among 2852 adolescents in primary and secondary school from November 2019 to January 2020 in Slovenia. 6-steps method of validation was used to test psychometric properties of the KIDSCREEN-27 scale. We checked descriptive statistics, performed a Mokken scale analysis, parametric item response theory, factor analysis, classical test theory and total (sub)scale scores.

**Results:**

All five subscales of the KIDSCREEN-27 formed a unidimensional scale with good homogeneity and reliability. The confirmatory factor analysis showed poor fit in user model versus baseline model metrics (*CFI* = 0.847; *TLI* = 0.862) and good fit in root mean square error (*RMSEA* = 0.072; *p*(*χ*^2^) < 0.001). A scale reliability was calculated using Cronbach's α (0.93), beta (0.86), G6 (0.95) and omega (0.93).

**Conclusions:**

The questionnaire showed average psychometric properties and can be used among adolescents in Slovenia to find out about their quality of life. Further research is needed to explore why fit in user model metrics is poor.

**Supplementary Information:**

The online version contains supplementary material available at 10.1186/s12955-022-01973-3.

## Background

Adolescence is the time between the ages of 10 and 19 when individuals have specific health and developmental needs [1]. The transition from childhood to adulthood often presents a challenging period with decline in emotional and mental well-being [2]. Thus, factors that influence emotional development of adolescents, such as personality changes, physical growth, and social and cognitive developments, must be taken into account [3]. Mental disorders often appear in this time, influencing later life. Investments in adolescents' mental well-being will bring great benefits in the following years and decades [4]. Adolescence provides an opportunity to develop positive mental health and wellbeing, and potentially reduce the costs of in health care caused by mental health disorders in adulthood [5].

Mental well-being compromises positive effects, evaluations of life, and absence of negative effects [6]. Many young people are facing everyday stress due to various stressors. Guo et al. [7] found that factors correlated to mental health are gender, physical activity, family economy, siblings, stress, satisfaction of self-appearance, quality of sleep, social trust, willingness to learn, as well as teachers and parent support. Adolescents spend many hours a day in school; thus, the school climate is of utmost importance for establishing mental well-being. Lester and Cross [8] found that factors like feeling safe, having a good connection to school and receiving peer support are protective factors of mental well-being, while connectedness to teachers is protective factor of mental well-being over the transition from primary to secondary school. Yet, Arabiat et al. [9] in their research in Jordan found that the parents' support is more important than school, and peer support is important for maintaining well-being in adolescence.

Social support is derived from family members, friends, and community, and is closely related to student's quality of life [10]. Social support is a protective factor to stress and contribute to one's well-being [11]. There is evidence that social support perception differs among gender, where female students receive more support from friends than from peers, family, and teachers. Male students receive less support from all sources [12]. Williams et al. [13] stated that social support requires the existence of social relationships with their structure, strength and type. Greater social support is linked to better health related quality of life among adolescents [14]. Moreover, lower social support for adolescents may contribute to emotional distress and internet addiction [15].

There are many methods available for measuring social support and quality of life (QoL) of adolescents, of these, the KIDSCREEN tools are most widely used [16, 17]. The KIDSCREEN-27 is a particularly useful instrument because it is easy to understand and use and because of the available volume of data for further comparisons. As the scale is widely used, it enables international comparison [18]. The KIDSCREEN-27 questionnaire is an instrument widely used for monitoring the quality of life of children and adolescents and relations with others. However, authors discussed issues with factor structure and item composition. Shannon et al. [19] used the scale among Irish children of low socio-economic status and reported that 5-factor model was an unacceptable fit. The modified 7-factor model was supported. Jervaeus et al. [20] reported acceptable goodness-of-fit values in 23 of 27 items. Also, the explained variance within each dimension was above the set criterion for all dimensions except Autonomy & Parent Relations. On the other hand, the scale achieved good levels of reproducibility, internal consistency, discriminatory power and construct validity among Brasilian adolescents [21]. The validation is a rigorous process and crucial for research in the field of health, social, and behavioural sciences. Scale evaluation can be onerous and is often unfamiliar to researchers because of the lack of graduate training on scale development and validation [22]. The COSMIN taxonomy suggested that for assessing measurement properties the following items must be tested: reliability, construct and content validity, reliability, measurement error and responsiveness [23]. Thus, factor analysis (structural validity) and Cronbach α (internal consistency) are not enough to perform psychometric analysis which will provide meaningful results [24]. Recently, advanced, and more user-friendly software-R has been developed that offers numerous packages for psychometric testing [25]. Researchers now have a program that ensures transparent and reproducible data. Yet, for beginners, writing script in R can be complicated and take much time, thus available scripts such as script by Dima [24] are highly welcome.

The aim of this study was the translation and validation of the KIDSCREEN-27 questionnaire which was used for measuring social support and quality of life of the school-aged adolescents in Slovenia following the 6-step psychometrics protocol in R.

## Methods

### Design

We conducted a cross-sectional study among adolescents from November 2019 to January 2020 in 16 primary and 9 secondary schools in Slovenia.

### The instrument

The KIDSCREEN questionnaire is available in three versions; the original long version consists of 52 items covering ten dimensions of QoL, a 27-item version covering 5 dimensions of QoL, and a 10-item index version [26]. The KIDSCREEN-27 measures five Rasch scaled dimensions: 1) physical well-being, 2) psychological well-being, 3) autonomy and parent relation, 4) peers and social support, and 5) school environments. The questionnaire was funded by the European Commission and developed within a European project "Screening and Promotion for Health-related Quality of Life in Children and Adolescents—A European Public Health Perspective" [27–29]. Each item was scored on a 5-point Likert scale ranging from 1 meaning "not at all" to 5 meaning "very much". Certain items (1, 9, 10 and 11) were reversed when scoring the questionnaire. Reverse scoring means that the numerical scoring scale runs in the opposite direction. The total KIDSCREEN score was calculated by summing up all the responses. Higher scores of KIDSCREEN indicated better QoL and social support. Before using the questionnaire, the prior permission to use was sought from the authors.

### Sample

According to the Statistical Office of the Republic of Slovenia [30] and the Ministry of Education, Science and Sport of the Republic of Slovenia [31] there were 454 primary and 182 secondary schools in Slovenia at the time of planning this project. We used random sampling based on which the expected sample included all available students from 22 primary schools (5% of all elementary schools) and 12 secondary schools (5% of all secondary schools) in Slovenia. Random sampling was performed using online Research Randomizer site. The required sample was calculated on the basis of data from the Statistical Office of the Republic of Slovenia [30], where it is evident that there were 176,898 enrolled students in elementary school and 74,021 in secondary school in school year 2016/2017. The envisaged final number of participants involved was determined by the size of the total population of students, degree of confidence and margin of error [32], which amounted to 384 students. The sample size was increased to avoid the risk of attrition and drop out during the study. Students who are under 10 years old and older than 19 years and those who are not included in the education system were excluded. Survey questionnaires were distributed to 3860 students in primary and 3107 students from secondary schools who consent to participation in the classroom where the classes took place.

The study was conducted in 16 primary and 9 secondary schools in different regions of Slovenia. Out of 6967 distributed questionnaires a total of 2972 were completed and returned which represents a response rate of 42.66%. A total of 1489 (50.10%) adolescents from the primary and 1483 (49.90%) from the secondary schools participated in the survey. Research involved primary school (from the 6th to the 9th grade) and secondary school students (from the 1st to the 4th grade). They were between 11 and 19 years old. Other characteristics of the sample are shown in the Table 1.

**Table 1:** Sample characteristics

	PS (*n* = 1489)	SS (*n* = 1483)
	*M (SD)*	*M (SD)*
Age	12.2 (1.5)	16.4 (1.3)

	*n (%)*	*n (%)*
Gender		
Female	768 (51.6%)	911 (66.8%)
Male	721 (48.4%)	452 (33.2%)
School year		
1st	0 (0.0%)	400 (29.3%)
2nd	0 (0.0%)	381 (28%)
3rd	0 (0.0%)	252 (18.5%)
4th	0 (0.0%)	311 (22.8%)
5th	264 (17.7%)	19 (1.4%)
6th	305 (20.5%)	0 (0.0%)
7th	266 (17.9%)	0 (0.0%)
8th	277 (18.6%)	0 (0.0%)
9th	377 (25.3%)	0 (0.0%)
Living environment		
With mother	1343 (90.7%)	1369 (91.9%)
With father	1204 (81.4%)	1205 (80.9%)
With sister/brother	1093 (73.9%)	1045 (70.2%)
With grandmother	316 (21.4%)	329 (22.1%)
With grandfather	214 (14.5%)	198 (13.3%)
Other	64 (4.3%)	59 (4.0%)

*PS* primary school students; *SS* secondary school students; *n* number of students; *M* Mean; *SD* standard deviation

The mean age among students in primary school was 12.2 (SD = 1.5) and 16.4 (SD = 1.3) among students in secondary school. Primary and secondary school students most frequently answered that they live with mother (PS: 1343, 90.7%; SS: 1369, 91.9%) or father (PS: 1204, 81.4%; SS: 1205, 80.9%). Students who answered “other” (PS: 48, 3.2%; SS: 55, 3.7%) described that they live either with godfather/godmother, great-grandfather/ great-grandmother, uncle/aunt, nephew, cousin, guardian, foster father/foster mother, stepfather/stepmother, or alone.

### Procedure

Data collection took place in the second half of 2019. We used a validated questionnaire KIDSCREEN-27 [33] to collect information on the perception of adolescents on support from family, friends, and teachers. Certain items (1, 9, 10 and 11) were reversed when scoring the questionnaire. The total KIDSCREEN score was calculated by summing up all the responses. Higher scores of KIDSCREEN indicated better QoL. The questionnaire was translated into Slovene following recommended guidelines [34]. The translation process included: (a) translation from English to Slovene by two independent native speakers of whom one knew about the instruments and concept under investigation and the other did not; (b) comparison of translated versions and (c) reaching a consensus; (d) back translation into the original language by two native speakers blind to the original version of the questionnaires; (e) comparison of a translated version and original version and (f) reaching a consensus among the involved translators.

The chosen institutions were contacted and approval for conducting the research was sought. On the agreed days, the questionnaires were distributed and collected. The data collection was performed in their classrooms by the teachers. The adolescents completed the questionnaires in a written (printed) version. It took approximately 20 min to complete the questionnaire.

The results of the research were analyzed using the programming language for statistical analysis R v 3.6.1. thus ensuring the reproducibility of the research that is often overlooked in nursing research.

### Analysis

The collected questionnaires were analyzed using the statistical program R [25]. A 6-step protocol for conducting psychometric analysis for detecting scale properties was used [21]. The basic descriptive statistics and plotting are often overshadowed by advanced methods. To prevent this, item-level descriptives were analyzed to detect outliers, out-of-range values, missing values, reverse-coded items, and item variations [21].

Examination of score distributions for further planned analyses was performed. Item properties were investigated via non-parametric and parametric item response theory (IRT). IRT is a psychometric theory of measurement that demonstrated reliability and validity of measurement [35]. A Mokken scale analysis (MSA) was performed to examine the homogeneity of items in the scale. MSA is a scaling technique used for ordinal data in scaling tests and questionnaires. It consists of two steps: (1) a selection algorithm that partitions a set of ordinal variables into scales (Mokken scales) and (2) methods to investigate assumptions of nonparametric IRT models [36]. Coefficients of homogeneity (H) for items in each subscale were calculated using mokken package [36]. Moreover, Automatic Item Selection Procedure (AISP) function from the Mokken package was used to explore possible dimensionality solutions at increasing thresholds [37].

The parametric IRT model can be used for further diagnosis of unidimensional scales fit for binary (Rasch model) or ordinal items (Rating scale model). In this study the Rating scale model as implemented in the eRm package [38] was used to calculate item-based characteristics. More specifically, the fit of actual responses and their logistic function was measured. According to Bond and Fox [39] the fit can be measured by putting more weight to responses from participants whose ability levels are closer to the item's difficulty level (infit) or putting more weight to extreme scorers (outfit). Item fit statistics (infit and outfit) indicate the model deviation. The mean squares of item fit should stay in the 0.5–1.5 range [40]. Additional to unidimensional IRT calculations, we also performed confirmatory Multidimensional IRT (MIRT) calculations at the item level using five factors of KIDSCREEN-27. The mirt package by Chalmers [41] was used to calculate corresponding RMSEA values.

Then the scale structure was explored using explanatory factor analysis (EFA) and confirmatory factor analysis (CFA), scale reliability using a classical test theory (CTT), and calculation and description of global scores. Factor analysis is a multivariate statistical procedure that reduces a large number of variables into a smaller set of variables (factors). It establishes dimensions between measured variables and latent constructs, and it provides construct validity evidence [42]. Factor analysis was conducted using package Psych [43] and Lavaan [44], which include various factor analysis options.

In the process of conducting CFA, different estimation methods were used: the root mean square error of approximation—RMSEA [45], comparative fit index—CFI [46], and Tucker–Lewis index—TLI [47, 48]. RMSEA is widely used as an absolute fit index that assesses properties of hypothesized model and compares it to a perfect model. CFI and TLI are incremental fit indices that compare the fit of a hypothesized model with that of a baseline model [49]. Parallel analysis method (PA) [50] and the Minimum Average Partial (MAP) [51] were considered when interpreting the data. PA is often used to determine the number of components to keep in a principal component analysis or factors to keep in an exploratory factor analysis. Moreover, MAP is used to determine the number of components, which focuses on the common variance in a correlation matrix. Very simple structure (VSS) was also considered as an alternative procedure for estimation of the optimal number of factors [52]. The sample size Bayesian information criterion (SABIC) was chosen as a model selection method [53]. CTT was conducted to test reliability for the item (sub)sets using Cronbach's α [54, 55] and other reliability indices such as ω, β, and Guttman's lambda 6—G6 [24, 56]. Dunn et al. [57] stated that calculating coefficient alpha is not enough to say that the measurement is reliable, thus they proposed calculation confidence intervals (CIs) by a method called bootstrapping, which involves repeated resampling with sample replacements to obtain an empirical distribution of an estimator [58]. Dima [24] also proposed calculation of omega, which makes more realistic assumptions than Cronbach's α alone.

### Ethics

Prior to the research, ethical approval was obtained from the Slovenian National Medical Ethics Committee (no. 0120-313/2019/13). Headmasters of schools were contacted and sought for the permission to carry out the research. Participants invited to the study were informed orally and in written form about the purpose of the research, instructions for compliance, confidentiality, anonymity, and volunteering, and the possibility of withdrawing from participation in any research phase. We informed them about the fact that we will use the obtained results exclusively for the purpose of the research. We adhered to the principles of the Helsinki Declaration on Biomedical Research in Human Beings [59], the Oviedo Convention [60], the Convention on the Rights of the Child [61], the Personal Data Protection Act [62], and of the Council and the principles of the Code of Medical Deontology of Slovenia [63]. Ethical principles of the Code of ethics in nursing and healthcare of Slovenia [64] were also considered and applied. Adolescents needed additional parental consent to participate in the study [65]. Additional information about the study were provided for students and parents. The contact information was also provided; thus, parent did have an opportunity to ask additional questions about the study. Both students younger than 16 years and parents signed the consent form. We store the consents to participate in the survey and survey questionnaires in locked rooms [66].

## Results

All 6 steps of psychometric testing are displayed in the following text.

### Step 1: Descriptive statistics

Firstly, response frequencies were checked for all items to show if items have sufficient variation to respondents. A minimum score for KIDSCREEN-27 was 30 and maximum 135. The mean value was 101.46 (SD = 18.43).

**Fig. 1:**
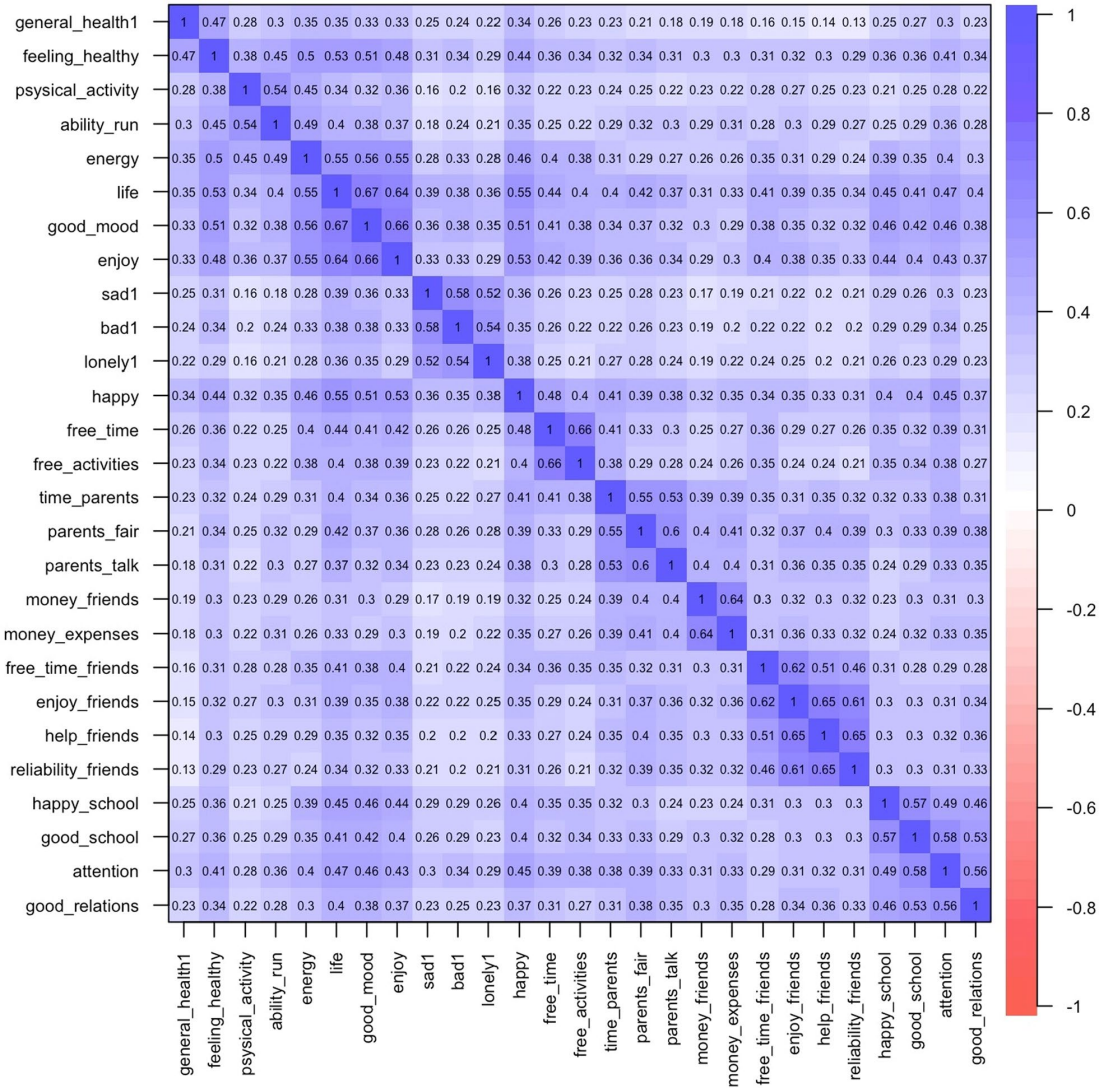
Correlation between KIDSCREEN-27 subscales

Secondly, subscales correlations were calculated to examine the power of mutual connections between subscales (Fig. 1).

There are no negative correlations nor outliers between items in the KIDSCREEN-27 subscales. Based on the statistical parameters and graphical data presentation, the KIDSCREEN-27 is constructed of five subscales.

### Step 2: Mokken scale analysis

The scale is homogeneous (H ≥ 0.40) for all five subscales in KIDSCREEN-27 (Table 2).

**Table 2:** Homogeneity coefficients for subscales of KIDSCREEN-27

Scale	*H (se)*
Physical well-being	0.450 (0.010)
Psychological well-being	0.479 (0.010)
Autonomy and parent relation	0.431 (0.010)
Peers and social support	0.664 (0.012)
School environment	0.574 (0.011)

*H* homogeneity coefficient; *se* standard errors

All items in KIDSCREEN-27 form a single scale at different homogeneity levels. The subscale physical well-being is unidimensional at the homogeneity level of 0.35, psychological well-being at 0.45, autonomy and parent relation at 0.55, peers and social support at 0.55, and school environment at 0.50 (Additional file 1).

### Step 3: Parametric item response theory (IRT)

A fit diagnosis was performed with the Rating scale model. Items infit and outfit present the extent to which responses fit logistic function for each item (Table 3).

**Table 3:** The mean square of item fit values

Scale	Infit		Outfit	
	Min	Max	Min	Max
Physical well-being	0.733	1.061	1.065	0.737
Psychological well-being	0.737	0.988	0.718	1.101
Autonomy and parent relation	0.749	1.045	0.780	1.066
Peers and social support	0.540	0.993	0.545	1.020
School environment	0.675	0.852	0.678	0.861

The KIDSCREEN-27 subscales have range of *infit* between 0.540 and 1.061, and *outfit* between 0.545 and 1.101. Additional to unidimensional IRT calculations, we also performed confirmatory multidimensional MIRT calculations at the item level using five factors. The *itemfit* RMSEA levels ranged between 0.011 and 0.027.

### Step 4: Factor analysis

**Fig. 2:**
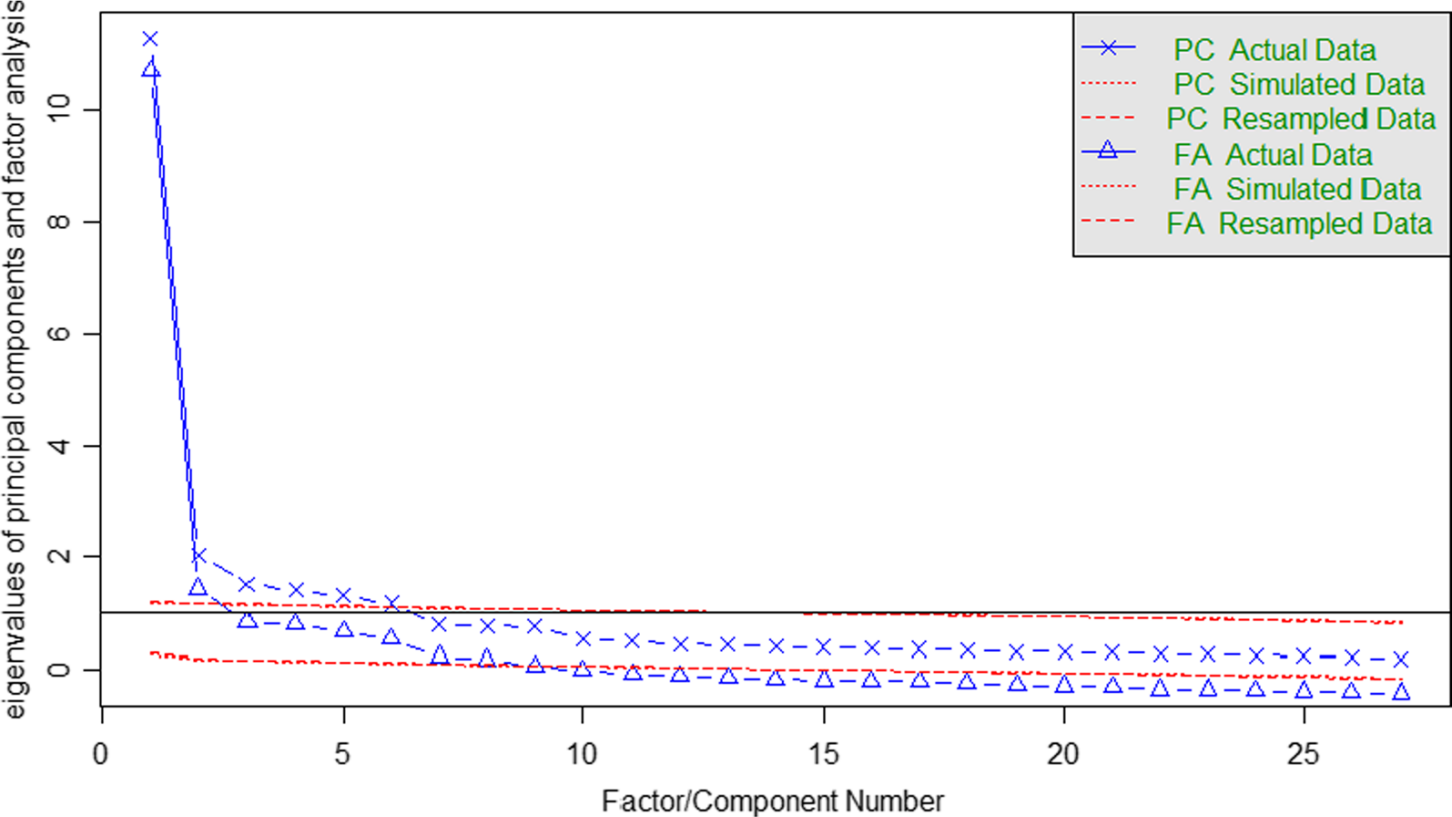
Parallel analysis scree plot

Conducted parallel analysis based on tetrachoric correlations suggested usage of 8 factors and 6 components for the KIDSCREEN-27 scale (Fig. 2).

**Fig. 3:**
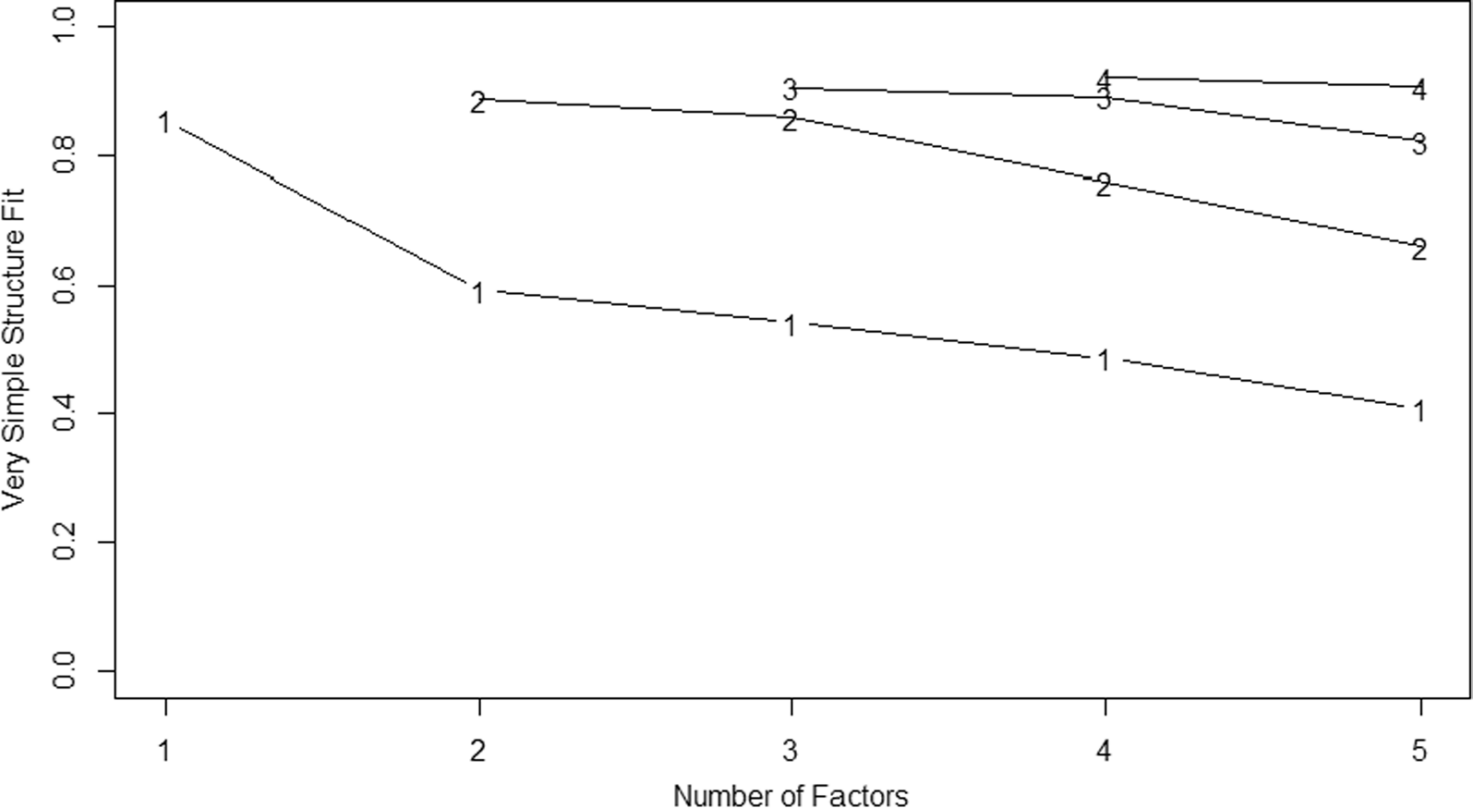
Very simple structure plot

Our results from the VSS analysis point to the optimal number of two factors, but both Velicer MAP and SABIC suggest a 5-factor structure (Fig. 3).

The CFA showed poor fit according to user model versus baseline model metrics (*CFI* = 0.847; *TLI* = 0.862) and good fit in root mean square error-based metric (*RMSEA* = 0.072; *p*(*χ*^2^) < 0.001).

### Step 5: Classical test theory (CTT)

In the step 5, a reliability for the KIDSCREEN-27 and sub-sets was calculated using Cronbach's α, beta, G6 lambda and omega (Table 4).

**Table 4:** Classical test theory values for KIDSCREEN-27 and subscales

	*α*	*β*	λ*	*ω*
KIDSCREEN-27	0.93	0.86	0.95	0.93
Physical well-being	0.78	0.67	0.75	0.79
Psychological well-being	0.85	0.72	0.86	0.84
Autonomy and parent relation	0.83	0.68	0.84	0.82
Peers and social support	0.87	0.84	0.85	0.87
School environment	0.82	0.79	0.78	0.82

*Guttman’s Lambda 6

### Step 6: Total (sub)scale scores

**Fig. 4:**
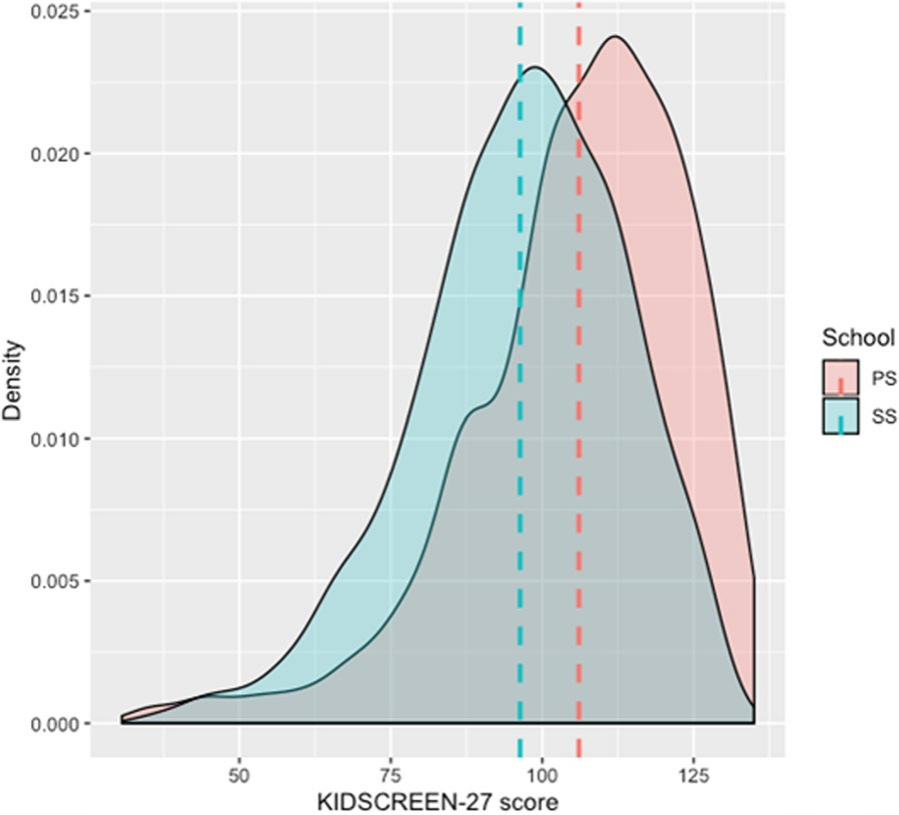
Data distribution of KIDSCREEN-27 scale

In the final step, data distribution was checked. Data in all subscales shows acceptable distribution (Fig. 4).

Summary statistics were calculated and are shown in Table 5. There were no outliers detected and all parameters show expected scores.

**Table 5:** Summary statistics

	*M*	*SD*
KIDSCREEN-27	101.23	18.46
Physical well-being	18.38	3.97
Psychological well-being	26.49	5.90
Autonomy and parent relation	26.26	5.76
Peers and social support	16.07	3.74
School environment	14.03	3.57

*M* mean; *SD* standard deviation

The Slovenian versions of the KIDSCREEN-27 scale achieved good validity, reliability, and psychometric properties.

## Discussion

The KIDSCREEN-27 questionnaire is instrument for monitoring QoL of children and adolescents to provide clarity on which factors from a wider social environment may contribute to children's or adolescents' mental well-being [19, 67]. The KIDSCREEN-27 was the first instrument for children that was tested in several countries [17–19, 28, 67–76] and in a large representative and multinational sample of children and adolescents in 13 countries (Austria, Czech Republic, France, Germany, Greece, Hungary, Ireland, Poland, Spain, Sweden, Switzerland, The Netherlands, and the United Kingdom) [77]. To our knowledge, this is the first published validation of the KIDSCREEN-27 among adolescents in Slovenia. The conducted analyses show that the Slovene version of the questionnaire has overall average psychometric properties for the use among adolescents. The questionnaire was translated following all the steps of the translation process. The questionnaire was than analyzed following a 6-step analysis in R: descriptive, non-parametric IRT, parametric IRT, factor analysis, classical test theory and total scores) [24].

According to the analysis, MSA identified one item that did not meet the criteria for ordinal scaling. The physical well-being subscale does not form a unidimensional scale at H ≥ 0.40. Fit diagnosis was performed using the Rating scale model. The KIDSCREEN-27 subscales have range of *infit* between 0.540 and 1.061, and *outfit* between 0.545 and 1.101. Similar results are reported by Vélez et al. [17]. Parallel analysis suggested 8 factors and 6 components, but both Velicer MAP and SABIC suggest a five-factor structure. The CFA showed average fit in the user model versus baseline model metrics (*CFI* = 0.847; *TLI* = 0.862) and good fit in root mean square error (*RMSEA* = 0.072; *p*(*χ*^2^) < 0.001). This might be since KIDSCREEN-27 questionnaire already measures QoL. CFA results of KIDSCREEN-27 among adolescents in Turkey [73] showed similar results (RMSEA = 0.07; CFI = 0.96). Poor results (RMSEA = 0.10; CFI = 0.91) were also reported in a study conducted among Chinese adolescents [72]. Another study suggested a modified 7-factor model, which is better at explaining the data than the original 5-factor model [19]. Ng et al. [72] agreed with a 7-factor model. However, studies conducted among 13 European countries and one conducted among Chilean adolescents [77] have found a 5-factor model acceptable. Since many studies show a variable number of underlying factors in different environments, usually resulting in a number of factors higher than five, one of the reasons for this might be related to the fact that the scale includes reversed items. As noted in a recent study by Zeng et al. [78], this might contribute to reduced internal consistency and change the factor structure of the scale.

The reliability and internal consistency of the KIDSCREEN-27 were calculated using Cronbach's α, beta, G6 and omega. Cronbach's α was 0.93 and is interpreted as acceptable [79–81]. Ng et al. [70] also reported acceptable internal consistency (0.84–0.89). Similar results were reported in a study among Turkish adolescents where Cronbach's α ranged between 0.78 and 0.84 [73]. They also reported that the dimension self-perception has lower Cronbach's α value (0.69), which confirms the results of Serbian (0.58) [70] and Iranian (0.60) [71] studies. In another study conducted among Irish children [19] Cronbach's α was below the recommended limit. Values of each subscale ranged from 0.56 to 0.74. Values in our study ranged from 0.78 to 0.93. Internal consistency was also evaluated using beta (0.86), lambda G6 (0.95) and omega (0.93), which other studies did not perform. Thus, additional comparisons cannot be performed.

Although our study was conducted following rigorous psychometric analysis and among large student sample, there are some limitations that are worth noting. The test–retest reliability was not conducted, because data was collected in a certain time frame. Moreover, the KIDSCREEN-27 is a self-reported questionnaire measuring social support, meaning that participants may give socially desirable answers. Thus, findings should be interpreted with caution. Although all invited schools had the same study protocol, most of them did not involve all students who are enrolled at their school. There may be a gap in how were students selected. Thus, due to a large sample of schools included in the study the authors of the study did not have the information on the strategies used to approach the students in the participating schools. Future studies are needed to evaluate the psychometric properties of the KIDSCREEN-27 scale and its clinimetric sensitivity and validity. Carrozzino et al. [82] recommended the Clinimetrics and its Clinimetric Patient-Reported Outcome Measures (CLIPROM) criteria which represent a step forward to the validation process of rating scales and daily clinical practice with patients.

## Conclusion

Overall, KIDSCREEN-27 is a valid and reliable questionnaire for measuring QoL of adolescents and the wider social factors that influence adolescents' mental well-being. The confirmatory factor analysis showed poor fit in user model versus baseline model metrics and good fit in root mean square error. This might be the consequence of having several latent variables in KIDSCREEN-27 questionnaire that are measuring different aspects of the QoL. The findings from this study add to the international understanding of the KIDSCREEN-27 scale and support the use of the Slovene translation of the scale. Although, scale underwent a rigorous process of psychometric testing following a 6-step analysis in R there are possibilities for improvements. A longitudinal design of a similar study would allow the observation of the temporal complexity in the KIDSCREEN-27 questionnaire results. Also, other study limitations should be considered when performing similar research.

## Supplementary Information

**Additional file 1**. Supplement 1: Mokken Scaling for KIDSCREEN subscales items.

## Data Availability

The datasets generated and analysed during the current study are not publicly available but are available from the corresponding author on reasonable request.
